# Comparison of the Analgesic Efficacy of Ultrasound-Guided Superficial Serratus Anterior Plane Block With Deep Serratus Anterior Plane Block in Patients Undergoing Modified Radical Mastectomy: A Randomized Clinical Trial

**DOI:** 10.7759/cureus.30828

**Published:** 2022-10-29

**Authors:** Indugumelli Jayadeep, Gnanasekaran Srinivasan, Adinarayanan Sethuramachandran, Lenin Babu Elakkumanan, Srinivasan Swaminathan, Prasanna Bidkar

**Affiliations:** 1 Anaesthesiology, Jawaharlal Institute of Postgraduate Medical Education & Research, Puducherry, IND; 2 Anaesthesiology and Critical Care, Jawaharlal Institute of Postgraduate Medical Education & Research, Puducherry, IND

**Keywords:** bupivacaine, postoperative pain, regional block, satisfaction score, pain score, analgesia, morphine, serratus plane block, radical mastectomy

## Abstract

Background

Acute postoperative pain after breast cancer surgery adversely affects recovery and is an independent predictor of chronic postsurgical pain in these patients. Serratus plane blocks have been found to provide analgesia to the anterior hemithorax. However, trials comparing superficial serratus plane block and deep serratus block in breast cancer surgery patients are sparse.

Methodology

A total of 74 female patients with American Society of Anesthesiologists physical status I and II scheduled for elective modified radical mastectomy for breast cancer were randomized into two groups. Group A patients received a superficial serratus plane block with 30 mL of 0.25% bupivacaine, and group B patients received a deep serratus plane block with 30 mL of 0.25% bupivacaine. Postoperatively, the Numerical Rating Scale (NRS) score was measured during the immediate postoperative period, after 30 minutes and at one, four, eight, 16, and 24 hours, as well as on the second and third day. After discharge, the NRS scores were recorded in the second and third weeks and then monthly once for three months. All patients received patient-controlled analgesia with intravenous (IV) morphine. The duration of analgesia, pain scores, and 24-hour morphine consumption were also noted.

Results

In group A, the mean duration of analgesia (hours) was 5.51 ± 1.42, whereas in group B the mean duration of analgesia (hours) was 6.69 ± 1.18 (p < 0.01). NRS scores for pain during rest at 12 and 16 hours and NRS scores for pain during cough at eight, 12, and 16 hours, as well as at the third month were significantly lower in group B. However, morphine consumption was comparable between the groups.

Conclusions

Deep serratus plane block was associated with better NRS scores for pain on rest and coughing and prolonged duration of analgesia after a modified radical mastectomy. We conclude that the deep serratus plane block provides superior and extended analgesia than the superficial serratus plane block after a modified radical mastectomy.

## Introduction

The mainstay of treatment in breast cancer is the surgical removal of the neoplasm. However, pain after breast cancer surgery poses significant problems in these patients. Acute postoperative pain can affect up to 60% of women undergoing mastectomy, with 10% of women developing chronic pain which can persist up to 6-12 months after surgery [1]. Acute post-mastectomy pain is an independent predictor of chronic postsurgical pain and adversely impacts the recovery of mastectomy patients [2]. Various regional blocks such as erector spinae block, paravertebral block, and intercostal block have been reported to manage acute postoperative pain following breast surgery.

The serratus anterior muscle can be easily visualized by sonography, and there are two potential compartments, namely, superficial and deep to the serratus anterior muscle. The description and clinical use of serratus anterior plane block (SAPB) were first done by Blanco et al. [3]. They described two potential spaces, namely, a superficial serratus plane between the posterior surface of latissimus dorsi and the anterior aspect of serratus anterior and a deep plane underneath the serratus muscles. The superficial SAPB aiming to anesthetize the lateral cutaneous branches of the thoracic intercostal nerves has been extensively studied and found to be effective in providing analgesia to the anterior and lateral parts of the chest wall along with the axilla after a radical mastectomy. Though the superficial SAPB has been found to provide analgesia of the anterior hemithorax, the deep SAPB was not widely studied in mastectomy patients with a dearth of prospective trials comparing the two plane blocks. Although studies have compared the deep SAPB with superficial SAPB [4-6], only a few studies have compared the above two blocks in breast cancer surgery patients.

In this study, we compared the effect of superficial and deep SAPB on immediate postoperative pain relief, consumption of analgesia, and pain scores in breast cancer patients who underwent a modified radical mastectomy. Our primary objective was to compare the postoperative pain scores using the Numerical Rating Scale (NRS) score at 30 minutes postoperatively during rest.

## Materials and methods

We included 74 female patients planned for a modified radical mastectomy during the period between April 2019 and March 2020 after obtaining clearance from the Institute Ethics Committee for human studies (IRB approval number: JIP/IEC/2017/0453). This trial was registered prospectively with the Clinical Trial Registry of India (CTRI/2019/03/018202). Only patients belonging to the American Society of Anesthesiologists (ASA) physical status classification I and II and above 18 years of age were included and those with an allergic history of local anesthetic drugs, coagulopathy, and infection at the site of intervention were excluded. Patients were enrolled after obtaining written informed consent. Randomization was done in the preoperative holding area, and 74 patients were randomized into two groups consisting of 37 patients each by block. Group A patients received superficial serratus anterior plane block, while group B patients received deep serratus anterior plane block. Group allocation was done by picking serially numbered opaque sealed envelopes by a resident who did not participate in the study, and blinding was not done. The primary objective was to study and compare the postoperative pain scores using the NRS score at 30 minutes postoperatively during rest. Secondary objectives were NRS pain scores at the immediate postoperative period, 30 minutes, first, fourth, eighth, 12th, 16th, 24th hours, second and third day, at the second and third week, followed by monthly once for the first three months postoperatively during rest and coughing. Secondary outcomes were the duration of analgesia in the postoperative period, postoperative analgesic consumption, procedure-related complications, and incidence of opioid-related side effects.

Patients were taught about self-assessment of pain using the NRS scale ranging from 0 to 10 (0 = no pain, 10 = worst pain), usage of patient-controlled analgesia, and their overall satisfaction using the Likert scale. All patients were premedicated with oral diazepam 5 mg on the previous night. After arriving at the operation theater, one hour before the scheduled time of the operation, all patients were shifted to the pre-anesthesia room, and peripheral venous access was secured using an 18-gauge intravenous (IV) cannula. Standard monitoring devices including non-invasive blood pressure, pulse oximetry, and five-lead electrocardiography were attached. All patients received premedication with IV midazolam 1 mg before the procedure. For performing block, all patients in both groups were placed in the lateral decubitus position placing the side to be blocked above with the arm abducted, and a linear transducer (8-13 MHz) ultrasonography (USG) (M-Turbo, Fujifilm, Sonosite Inc, USA) probe was used under aseptic precautions by trained anesthesiologists. The USG probe was placed over the mid-clavicular region of the thoracic cage on the operating side in a sagittal plane. By counting the ribs inferiorly and laterally in the mid-axillary line, the fourth and fifth ribs were identified. In group A, patients received superficial SAPB as follows: the latissimus dorsi and serratus muscle were identified, and after infiltration of the skin with 2% lignocaine, a 22-gauge echogenic needle was introduced in-plane with respect to the USG probe from the caudal to cranial direction till the tip of the needle was placed between the two muscles and the position was confirmed by injecting saline. After confirming the plane between the two muscles with hydrodissection by saline, 30 mL of 0.25% bupivacaine was injected. In group B patients, deep SAPB was given by injecting 30 mL of 0.25% bupivacaine in the fascial plane between the serratus anterior and the intercostal muscles.

The sensory level of the block was assessed by an anesthesiologist not involved in the study, with a pin-prick sensation every five minutes in each dermatomal distribution from T2 to T6 in the mid-clavicular, mid-axillary, and mid-scapular lines. The total number of dermatomes with no pain to pinprick compared with the opposite side was noted. The block was considered a failure if the pinprick sensation did not decrease in any segment for up to 30 minutes. Block-related complications such as pneumothorax, hypotension, and vascular injury were noted.

After confirming the sensory level of the block, all patients in both groups were shifted to the operating room, and general anesthesia was induced using a standard protocol. All patients were shifted to the post-anesthesia care unit at the end of surgery.

The postoperative pain was assessed using the NRS scale (0-10, 0 = no pain, 10 = worst pain imaginable) by an independent observer not involved in the study. The vital parameters and pain scores at rest and on coughing were noted at the following timelines: immediate postoperative period, 30 minutes, first, fourth, eighth, 12th, 16th, and 24th hours. All patients in both groups received patient-controlled analgesia with IV morphine in the postoperative period for 24 hours using the following settings: demand dose of 1 mg morphine with a lockout time of 15 minutes. The time from the administration of the serratus plane block to the time of administration of the first dose of patient-controlled morphine by the patient in the postoperative period was considered as the duration of analgesia. The total morphine consumed in the first 24-hour postoperative period was also noted. Other parameters such as postoperative nausea and vomiting (PONV) and complications were noted. NRS scores for pain were assessed daily until the third day and thereafter at the second and third week and monthly for three months over the telephone. The overall satisfaction with analgesia was assessed using the Likert scale.

Okmen et al. found reduced Visual Analog Scale (VAS) scores (1.8 ± 0.76) with serratus plane block compared to conventional analgesia in thoracotomy patients [7]. By expecting a difference in the mean VAS scores at 24 hours during rest by one with a standard deviation of 1.5, we calculated a sample size of 37 patients in each group to achieve a power of 80% and a level of significance of 5%. Statistical analysis was done using SPSS version 19 (IBM Corp., Armonk, NY, USA). During follow-up in the postoperative period, one patient in group B received a different analgesic agent, which violated the protocol. After the exclusion of that patient, we analyzed 37 patients in group A and 36 patients in group B. Comparison of age and duration of surgery between groups A and B was done using the independent t-test. Weight and height comparison was done using the Mann-Whitney U test, and ASA physical status classification was compared using the chi-square test.

## Results

Among the demographic parameters, age, height, weight, ASA physical status classification, and duration of surgery were not statistically significant, as shown in Table 1.

**Table 1:** Demographic characteristics of the two groups. SD: standard deviation; IQR: interquartile range; ASA PS: American Society of Anesthesiologists physical status

Parameters		Group A (N = 37)	Group B (N = 36)	P-value
Age (years) [mean ± (SD)]		51.16 ± 8.59	54.56 ± 9.98	0.12
Height (cm) [median (IQR)]		155(5)	154.5 (5)	0.62
Weight (kg) [mean ± (SD)]		50(15)	50 (15)	0.38
ASA PS class	I	23	26	0.36
	II	14	10	
Duration of surgery (hours)		2.73 ± 0.60	2.65 ± 0.68	0.64

NRS pain scores at rest analyzed using two-way repeated measures of the analysis of variance method were significantly higher at the following timelines: in group A - one hour, 12 hours, and 16 hours, as shown in Table 2. NRS pain scores on coughing were significantly higher in group A at the following timelines: eight hours, 12 hours, 16 hours, and the third month, as shown in Table 3.

**Table 2:** NRS pain scores at rest in the two groups. *p < 0.05 is statistically significant. NRS: Numerical Rating Scale

Time points	Group A (N = 37)	Group B (N = 36)	P-value
Immediate postoperative	6.57 ± 0.6	6.5 ± 0.8	0.94
30 minutes	5.9 ± 0.86	5.8 ± 0.89	0.59
60 minutes	3.6 ± 1.13	4.2 ± 0.93	0.01*
4 hours	5.23 ± 1.23	5 ± 1.30	0.24
8 hours	3.8 ± 1.0	3.7 ± 0.91	0.46
12 hours	3.76 ± 1.0	3.08 ± 0.90	0.00*
16 hours	3.54 ± 1.32	2.86 ± 0.83	0.01*
24 hours	2.95 ± 1.0	2.81 ± 0.88	0.54
Day 2	2.41 ± 0.49	2.14 ± 0.63	0.05
Day 3	1.78 ± 0.9	1.64 ± 0.63	0.53
Week 2	0.78 ± 0.34	0.47 ± 0.26	0.23
3rd week	0.64 ± 0.44	0.32 ± 0.20	0.43
1st month	0.03 ± 0.17	0.03 ± 0.17	1.0
2nd month	0.00	0.00	1.0
3rd month	0.00	0.00	1.0

**Table 3:** NRS pain scores on coughing in the two groups. *p < 0.05 is statistically significant. NRS: Numerical Rating Scale

Time points	Group A (N = 37)	Group B (N = 36)	P-value
Immediate postoperative	8.3 ± 0.46	8.4 ± 0.60	0.34
15 minutes	8.3 ± 0.46	8.4 ± 0.60	0.34
30 minutes	7.62 ± 0.98	7.58 ± 0.90	0.86
45 minutes	5.97 ± 1.0	6 ± 1.18	0.68
60 minutes	5.16 ± 1.32	5.56 ± 1.18	0.18
4 hours	7.14 ± 1.29	6.61 ± 1.67	0.14
8 hours	5.43 ± 1.25	4.8 ± 1.03	0.04*
12 hours	5.16 ± 1.14	4.25 ± 1.02	0.01*
16 hours	4.6 ± 1.31	4.0 ± 0.86	0.01*
24 hours	4.2 ± 0.93	3.9 ± 0.92	0.13
Day 2	3.54 ± 0.50	3.31 ± 0.74	0.12
Day 3	2.95 ± 0.70	2.81 ± 0.71	0.39
2nd week	1.71 ± 0.43	1.32 ± 0.31	0.17
3rd week	1.43 ± 0.32	1.10 ± 0.45	0.23
1st month	1.15 ± 0.55	0.97 ± 0.71	0.26
2nd month	0.94 ± 0.42	0.70 ± 0.63	0.07
3rd month	0.94 ± 0.42	0.67 ± 0.64	0.04*

The postoperative morphine consumption was not statistically significant between the groups. However, the duration of analgesia was significantly longer in group B, as shown in Table 4. The dermatome level spread between the groups was comparable. Out of 37 patients in group A, 27 expressed “extreme satisfaction” and 10 expressed “satisfaction” on the Likert scale. In group B, all 36 patients expressed “extreme satisfaction” (p = 0.001).

**Table 4:** Comparison of analgesic efficacy. *p < 0.05 is statistically significant. SD: standard deviation

Parameters	Group A (N = 37)	Group B (N = 36)	P-value
Duration of analgesia (hours) [mean ± (SD)]	5.51 ± 1.42	6.69 ± 1.18	0.01*
Morphine consumption (mg) [mean ± (SD)]	5.24 ± 0.69	5.15 ± 0.64	0.56

## Discussion

Our study results show that deep SAPB is associated with lesser pain scores for acute postoperative pain and chronic pain three months after surgery. Further, deep SAPB prolonged the duration of analgesia significantly.

Breast cancer surgery involves extensive dissection and resection of major muscles and nerves, causing severe pain postoperatively which may progress to chronic pain. Serratus plane block was first described by Blanco et al. as a regional analgesic technique to provide analgesia to the hemithorax [3], and it was studied extensively in breast cancer surgery patients [8,9]. Blanco et al. described two potential compartments, namely, superficial and deep to serratus muscle, and observed that local anesthetic solution injection into both of these spaces provided durable regional anesthesia [3]. Piracha et al. studied deep serratus plane block in patients with post-mastectomy syndrome, in which local anesthetic solution was injected into the plane between the serratus muscle and external intercostals [10]. Though there are previous studies comparing superficial and deep serratus plane blocks, our study differs from those studies in the following aspects: chronic pain at rest and coughing for up to three months was observed and postoperative patient-controlled morphine consumption was also noted.

Abdallah et al. found that deep serratus plane block was associated with non-inferior but not superior pain scores in ambulatory breast surgery [11]. Edwards et al. on comparing the above two techniques in mastectomy found that the deep block patients had lower pain scores at 12 hours postoperatively [4]. In our study, we observed that deep SAPB was associated with significantly lower NRS scores during rest at 12 hours, 16 hours, and on day two. Similarly, deep block patients had lower NRS scores during coughing at eight hours, 12 hours, and 16 hours. In contrast to the above two studies, we also studied NRS scores for chronic pain and found that patients who received deep serratus block had lower NRS pain scores on coughing in the third month. Regis et al. studied the effect of deep serratus block on chronic pain at three months after breast conservation surgery and found that 69% of patients did not have persistent pain at three months [12]. Our study compared chronic pain scores between superficial and deep serratus block, though we did not use questionnaires to assess chronic pain and functional status.

Although the duration of analgesia was significantly prolonged with deep SAPB, the total patient-controlled morphine consumption in the postoperative period was insignificant between the two groups. This contrasted with the findings of Edwards et al. who observed that deep serratus block patients required lesser oral morphine postoperatively [4]. Our study differed from the above study by using intravenous patient-controlled morphine. Piracha et al. in a brief report described four cases in which the deep serratus plane block provided a prolonged duration of analgesia [10]. Mazzinari et al. in a randomized controlled trial compared deep serratus plane block with conventional analgesia and found that serratus plane block significantly prolonged the first rescue analgesic dose request [13]. Our study results mirrored the results of these two studies regarding the duration of analgesia.

Bhoi et al in a case series including 20 patients compared the superficial and deep blocks and found no significant difference in NRS scores and fentanyl consumption [14]. This was contrary to our trial results, where we observed that deep SAPB significantly reduced NRS scores in the immediate postoperative period although postoperative morphine consumption was not significant.

All 36 patients in group B expressed “extreme satisfaction” on the Likert scale compared to group A (27 out of 37 patients), which was statistically significant. This was in contrast to the study by Edwards et al. who found no significant difference in the satisfaction score between the superficial and deep blocks [4]. None of our patients had PONV, and no block failure was noted. No procedure-related complications were noted.

Our study had certain limitations. First, the sample size was smaller. Second, we did not perform postoperative dermatome mapping which would have helped in assessing the spread of the block over time. Third, we did not record the hemodynamic response to surgical incision. Fourth, NRS pain scores may not be optimal to study chronic pain, and further trials are needed to study the impact of serratus plane block on chronic pain and functional status after breast cancer surgery.

## Conclusions

Deep SAPB is associated with better NRS scores for pain on rest and coughing in the initial 24 hours of the postoperative period after a modified radical mastectomy. Further, deep serratus block provides a longer duration of analgesia and better patient satisfaction. We conclude that the deep serratus plane block provides superior and extended analgesia than the superficial serratus plane block after modified radical mastectomy for breast cancer. Further trials are required to study the effect of serratus plane blocks on chronic pain and functional assessment.
